# Macrophage adaptation to hypoxia: metabolism, migration, and phagocytosis

**DOI:** 10.3389/fcimb.2025.1706664

**Published:** 2025-12-12

**Authors:** Lu Yuan, Tiemen Mellema, Gésinda I. Geertsema-Doornbusch, Henny C. van der Mei

**Affiliations:** Biomaterials and Biomedical Technology, University of Groningen and University Medical Center Groningen, Groningen, Netherlands

**Keywords:** bacteria, host-pathogen interaction, oxidative stress, periodontitis, inflammation

## Abstract

**Background:**

Hypoxia is a hallmark of many diseases, including periodontitis, where it influences the immune cell behavior. In low-oxygen conditions, macrophages shift toward glycolytic metabolism, altering their phenotype and function, However, it remains unclear how these functional changes affect the interaction between macrophages and oral bacteria in the hypoxic environment of infectious tissue.

**Methods:**

In this study, which focused on periodontitis, we addressed this gap by investigating how a physiologically relevant low oxygen tension (2% O_2_) compared with normoxia (20% O_2_) modulates the metabolism, morphology, migration, and interaction of macrophages with both commensal and pathogenic oral bacteria.

**Results:**

Hypoxia activated the hypoxia-inducible factor-1 alpha (HIF-1α) signaling pathway, induced glycolytic metabolism, reduced proliferation, and led to a rounded morphology with amoeboid migration characteristics. Despite reduced mobility, hypoxic macrophages maintained their phagocytic capacity and effectively limited the intracellular proliferation of *Streptococcus oralis* and *Porphyromonas gingivalis*. Hypoxia also altered the cytokine profiles, with increased interleukin-1 beta (IL-1β) and IL-10, reduced tumor necrosis factor alpha (TNF-α), and enhanced reactive oxygen species production.

**Conclusion:**

These findings highlight the plasticity of macrophages in adapting to low-oxygen environments and underscore their potential role in host defense and inflammation resolution in periodontal disease. The modulation of these responses may inform novel therapeutic approaches targeting hypoxia-associated immune dysfunction.

## 1 Introduction

Bacterial infections are closely related to the course and the pathogenesis of numerous human diseases, exerting severe effects on both localized tissues and systemic health (Zhang et al., 2023). Infections within the oral cavity have garnered attention due to their potential to mediate chronic inflammatory conditions such as periodontal diseases. Periodontal diseases affect both the soft and hard periodontal tissues, leading to progressive tissue destruction and, ultimately, tooth loss (Van Dyke and Sima, 2020; Saleh et al., 2025). Globally, severe periodontal diseases affect approximately 19% of individuals over the age of 15, accounting for over 1 billion cases worldwide (World Health Organization, 2022). The pathogenesis of periodontal disease involves a complex interplay between microbial biofilms and the host immune response. In this context, the immune response mediates periodontal tissue destruction through an uncontrolled inflammatory response, which targets the host’s own cells and tissues, causing persistent redness, swelling, and tissue damage. Among the various immune cell types involved, macrophages play a crucial role in the local immune milieu (Sima and Glogauer, 2013). They participate in pathogen recognition, cytokine production, phagocytosis, and tissue remolding. However, their function is highly dependent on the surrounding microenvironment, which in periodontitis is markedly altered. One of the most defining features of the inflamed periodontal microenvironment is hypoxia, a state of reduced oxygen availability. This is due to the increased oxygen consumption by infiltrating immune cells, the overgrowth of microbial biofilms, and a compromised blood flow (Bozyel et al., 2025). Clinical studies have reported that the oxygen levels in the periodontal pockets of disease sites can become approximately 2% (Müller-Heupt et al., 2023).

This drop in oxygen tension has significant implications for immune cell functions. Hypoxia leads to the stabilization of the hypoxia-inducible factor (hypoxia-induciblefactor-1 alpha (HIF-1α), a transcription factor that mediates cellular adaptation to low-oxygen environments. HIF-1α activation drives a metabolic shift of macrophages toward an enhanced rate of glycolysis to sustain adenosine triphosphate (ATP) production. This process is mediated by glucose transporters under the regulation of the hypoxia-inducible factor (HIF) pathway (Kierans et al., 2023). These HIF-dependent metabolic alterations induce significant changes in macrophage metabolism, promoting a switch toward a pro-inflammatory phenotype, which supports the production of antimicrobial effectors including reactive oxygen or nitrogen species and antimicrobial peptides (Rankin and Giaccia, 2016; Taylor and Scholz, 2022). Conversely, the macrophage anti-inflammatory phenotype often uses oxidative phosphorylation for ATP generation (Russell et al., 2019).

Despite the clear biological importance of hypoxia in inflamed tissue, much of our current understanding of the functions of macrophages is based on studies performed under normoxic conditions, which do not reflect the physiological reality of diseased sites. There is a growing need to understand how oxygen tension, particularly at clinically relevant hypoxic levels such as 2% O_2_, affects the behavior of macrophages.

In this study, we aimed to explore the function adaptation of macrophages to a 2% O_2_ environment, representing the hypoxic conditions commonly found in periodontal pockets. Using a murine macrophage cell line (i.e., J774A.1), we evaluated how hypoxia influences key cellular processes such as proliferation, metabolic activity, morphology, migration, cytokine secretion, and phagocytic response to both commensal (*Streptococcus oralis*) and pathogenic (*Porphyromonas gingivalis*) bacteria. Live cell imaging and bacterial co-culture assays were employed to assess dynamic cell behaviors and antimicrobial efficacy. By characterizing the responses of macrophages under physiologically relevant hypoxia, this work provides insights into how oxygen tension modulates the innate immunity in inflamed tissues and may inform the development of targeted therapies for periodontal and other hypoxia-associated diseases.

## 2 Materials and methods

### 2.1 Bacterial and macrophage culture conditions

*S. oralis* ATCC 35037 and *P. gingivalis* ATCC 33277 were purchased from the American Type Culture Collection (ATCC, Manassas, VA, USA). The bacteria were streaked on a blood agar plate from a frozen stock. *S. oralis* was grown in a humidified aerobic incubator (5% CO_2_) at 37°C overnight, while *P. gingivalis* was grown in an anaerobic chamber (5% CO_2_, 85% N_2_, and 10% H_2_) at 37°C for 5–7 days. One colony of *S. oralis* was inoculated in 10 ml brain heart infusion (BHI; 37 g L^−1^) (ThermoFisher, Waltham, MA, USA) broth supplemented with yeast extract (1 g L^−1^) (Becton Dickinson, Franklin Lakes, NJ, USA) and incubated in the aerobic incubator with 5% CO_2_ at 37°C for 24 h. Several colonies of *P. gingivalis* were inoculated in 10 ml BHI broth with yeast extract and incubated in the anaerobic incubator for 24 h. Thereafter, 4 ml of the pre-cultures was transferred into 80 ml of fresh media and cultured for another 16 h. Bacterial cultures were harvested by centrifugation (6,500 × *g*, 5 min, 10°C), washed twice with phosphate-buffered saline (PBS) (5 mM KH_2_PO_4_, 5 mM K_2_HPO_4_, and 150 mM NaCl, pH 7.0), and resuspended in 10 ml PBS. The *S. oralis* suspension was sonicated for 30 s in an ice water bath. The concentrations of *S. oralis* and *P. gingivalis* were determined by enumeration in a Bürker–Türk counting chamber, after which the suspensions were diluted to the concentrations required in the experiment.

Murine macrophage J774A.1 (TIB-67, ATCC) was purchased from the American Type Culture Collection. The macrophages were cultured in Dulbecco’s modified Eagle’s medium containing 4.5 g L^−1^ glucose (DMEM-HG; Gibco, Carlsbad, CA, USA) and supplemented with 10% (vol/vol) fetal bovine serum (FBS) (Gibco, Carlsbad, CA, USA) in a humidified incubator (5% CO_2_) at 37°C. The cells were detached from the culture flask surface with a cell scraper after reaching 70%–90% confluency. The macrophages were collected by centrifugation (1,000 rpm, 5 min) and the pellet resuspended in fresh culture medium. The cells were stained with trypan blue to discriminate live and dead cells, and the cell density was determined using a Bürker–Türk counting chamber, after which the suspension was diluted to the concentration required in the experiment.

### 2.2 Metabolic activity, morphology, and glycolysis of macrophages in hypoxic and normoxic conditions

The metabolism of the macrophages exposed to hypoxia (2% O_2_, 5% CO_2_, and 93% N_2_) (CellXpert C170i; Eppendorf, Hamburg, Germany) or normoxia (20% O_2_, 5% CO_2_, and 75% N_2_) (CellXpert C170; Eppendorf, Hamburg, Germany) for 24 and 72 h was determined with the XTT assay (PanReac AppliChem, Darmstadt, Germany). Briefly, 100 µl of the macrophage suspension (5 × 10^4^ ml^−1^) was added to each well of a 96-well plate and incubated under the hypoxic or normoxic condition for 24 or 72 h. The metabolic activities of the macrophages were measured following the user guide. After 24 or 72 h, 50 µl of an XTT reagent was added to the well and incubated for another 2 h under the hypoxic or normoxic condition. Subsequently, the absorbance was measured using a plate reader (Biotek Synergy H1; Agilent, Waldbronn, Germany) with dual wavelengths at 450 and 660 nm as the reference. Wells with only the culture media and activated XTT reagent were used as blank controls. The cellular morphologies were analyzed with phase-contrast microscopy before adding the XTT reagent. Glycolysis of the macrophages cultured under 2% or 20% O_2_ at 37°C were measured by the extracellular acidification in the glycolysis process. Macrophages (5 × 10^4^ cells/well) were seeded in a 96-well plate and cultured under 2% or 20% O_2_ with 5% CO_2_ at 37°C for 18 h. Prior to the glycolysis assay (ab197244; Abcam, Cambridge, UK), the cells were exposed to a CO_2_-free incubator at 37°C for 3 h. Subsequently, the fluorescence intensity was measured for 1.5 h at time intervals of 2 min in a respiration buffer containing the glycolysis reagents and the glycolysis rate was calculated.

### 2.3 HIF-1α immunofluorescence staining and fluorescence intensity analysis

Macrophages cultured in hypoxic and normoxic conditions for 24 h were fixed with 4% formaldehyde for 15 min, washed twice with PBS, permeabilized with 0.2% Triton X-100 for 5 min at room temperature, and washed again with PBS. After incubation with 1% bovine serum albumin dissolved in PBS (1% PBSA) for 30 min, the cells were incubated with a primary antibody, anti-HIF-1α (ab178483; Abcam, Cambridge, UK), with dilution of 1:500 in 1% PBSA at 4°C for 24 h. Thereafter, the cells were washed twice with PBS at room temperature and stained using a secondary antibody, goat anti-rabbit IgG Alexa Fluor^®^ 488 (ab150077; Abcam, Cambridge, UK), with dilution of 1:1,000 in 1% PBSA for 1 h, washed with PBS, cross-stained with DAPI (1:100 in PBSA) for another 30 min in the dark at room temperature, and washed again with 1% PBSA and PBS. Confocal laser scanning microscopy (CLSM) (Leica Upright Stellaris 5; Leica, Wetzlar, Germany) was used for imaging. The same settings of the CLSM were applied for all samples. Briefly, 405- and 488-nm lasers with emission wavelength ranges of 420–470 nm and 490–548 nm were applied for the DAPI and Alexa Fluor 488 dyes, respectively. Fiji software (National Institutes of Health, Bethesda, MD, USA) was used for the calculation of the fluorescence intensity of HIF-1α in the cell nucleus (Schneider et al., 2012). Briefly, DAPI channel images were transformed into binary images, which were used as masks for HIF-1α fluorescence intensity calculation with the Fiji function of the image calculator, followed by measurement of the fluorescence intensity of the HIF-1α channel. The fluorescence intensities of HIF-1α were quantified by subtracting the background from the measured intensities. The background was determined using images of the macrophages stained with secondary antibodies alone.

### 2.4 Co-culture of macrophages and bacteria

*S. oralis* and *P. gingivalis* were diluted to a final concentration of 10^7^ ml^−1^ in the optimized co-culture medium (80% DMEM-LG + 10% FBS + 10% BHI) (**Supplementary Figure S1**). The bacterial suspension (10^7^ ml^−1^) was added to the wells of 12-well plates (1 ml per well). Bacteria were incubated in 5% CO_2_ at 37°C for 1 h to let them adhere to the polystyrene surface of the well. Thereafter, the non-adherent bacteria were removed and 1 ml of the fresh co-culture medium with or without macrophages (10^5^ ml^−1^) was added. To study the macrophage phagocytosis in hypoxic and normoxic conditions, the plates were placed in an incubator with 2% and 20% O_2_, respectively, at 37°C at predetermined time points.

### 2.5 Migration of macrophages under hypoxic and normoxic conditions

Macrophages with or without bacteria in the co-culture were live-tracked using a microscope (CellDiscoverer 7; Zeiss, Oberkochen, Germany) with environmental controls (5% CO_2_, 37°C, 2% O_2_ or 20% O_2_). Images were acquired at 5-min intervals for 2 h (×400 magnification and 2 × 2 binning) with bright-field and phase-contrast microscopy. Fiji software was used to analyze the live images, with the region of interest (ROI) manager and the track mate function used for the cellular morphology and the migration analysis, respectively (Ershov et al., 2022).

### 2.6 Intracellular bacteria in macrophages

After incubation of the co-cultures in hypoxic and normoxic conditions for 2 and 24 h, the growth medium was removed and refreshed with 1 ml co-culture medium containing gentamicin (100 µg ml^−1^), then incubated in the hypoxic or normoxic condition (5% CO_2_, 37°C) for 1 h to kill the extracellular bacteria (Cecotto et al., 2022). After 1 h, the adherent macrophages and bacteria were washed twice with 1 ml PBS, 0.6 ml of demineralized water was added, and the macrophages collected using a cell scraper. The wells were washed with demineralized water and 4 ml of the macrophage suspension was sonicated in an ice water bath for 30 cycles (1-s sonication, 2-s pause). Subsequently, the suspensions were serially diluted and plated on agar plates. Suspensions containing *S. oralis* were plated on BHI agar and incubated at 37°C (5% CO_2_, 95% air) for 24 h before counting the colony-forming units (CFU). *P. gingivalis* was plated on blood agar plates and incubated in an anaerobic chamber for 7 days before CFU counting. In a parallel study to investigate whether intracellular bacteria can survive and replicate in macrophages, after 2 h of co-culture, the plate was incubated with 100 µg ml^−1^ gentamicin for 1 h to kill the extracellular bacteria and then replaced with antibiotic-free co-culture media for an additional 21 h. Thereafter, the macrophages were washed, collected, sonicated, plated on agar plates, and counted following the steps described previously.

### 2.7 Fluorescence staining and imaging of macrophages

Macrophages after 2 and 24 h co-culture with or without bacteria were fixed with 4% formaldehyde for 15 min, washed with PBS twice, permeabilized with 0.2% Triton-X100 for 5 min, incubated with 1% PBSA for 5 min, stained with phalloidin–fluorescein isothiocyanate (FITC) (1:1,000 in PBSA) and DAPI (1:100 in PBSA) for 30 min in the dark at room temperature, and washed with 1% PBSA and PBS. CLSM (Leica Upright Stellaris 5; Leica, Wetzlar, Germany) was used for imaging of the intracellular bacteria in macrophages.

### 2.8 Intracellular reactive oxygen species

The intracellular reactive oxygen species (ROS) of the macrophages were detected using a 2′7′-dichlorodihydrofluorescein diacetate probe (DCFH-DA; Sigma-Aldrich, Amsterdam, Netherlands). For macrophages with or without bacteria, after culturing under different oxygen levels for 2 h, the culture medium was refreshed with a mixture of dyes (5 µg ml^−1^ of DCFH-DA and 10 µg ml^−1^ of Hoescht 33342 in PBS) and then incubated at 37°C and 5% CO_2_ with 2% or 20% O_2_ for 30 min. The cells were then washed twice with PBS and imaged with CLSM. Fiji software was used to quantify the fluorescence intensity, with Hoescht-stained nuclei used to normalize the intracellular ROS level per cell.

### 2.9 Cytokine secretion

The cytokine (IL-1β, IL-6, IL-10, and TNF-α) concentrations in the medium secreted by the macrophages were determined using enzyme-linked immunosorbent assay (ELISA). The supernatant of the macrophages cultured with and without bacteria for 2 h under 2% and 20% O_2_ was collected and centrifuged at 800 × *g*. The cytokine concentrations in the supernatant were determined using ELISA kits (Proteintech, Planegg-Martinsried, Germany).

### 2.10 Statistical analysis

All experiments were conducted in triplicate, and the results are represented as the mean ± standard deviation (SD). Student’s *t*-test was used for the comparison of two groups, while one-way analysis of variance (ANOVA) followed by Tukey’s test was used for the comparison of multiple groups. Two-way ANOVA was used to assess the differences between the interaction factors on GraphPad Prism 10.3.0. Statistical significance was considered at *p* < 0.05.

## 3 Results

### 3.1 Effects of hypoxia on macrophage HIF-1α activation, metabolism, and proliferation

**Figure 1:**
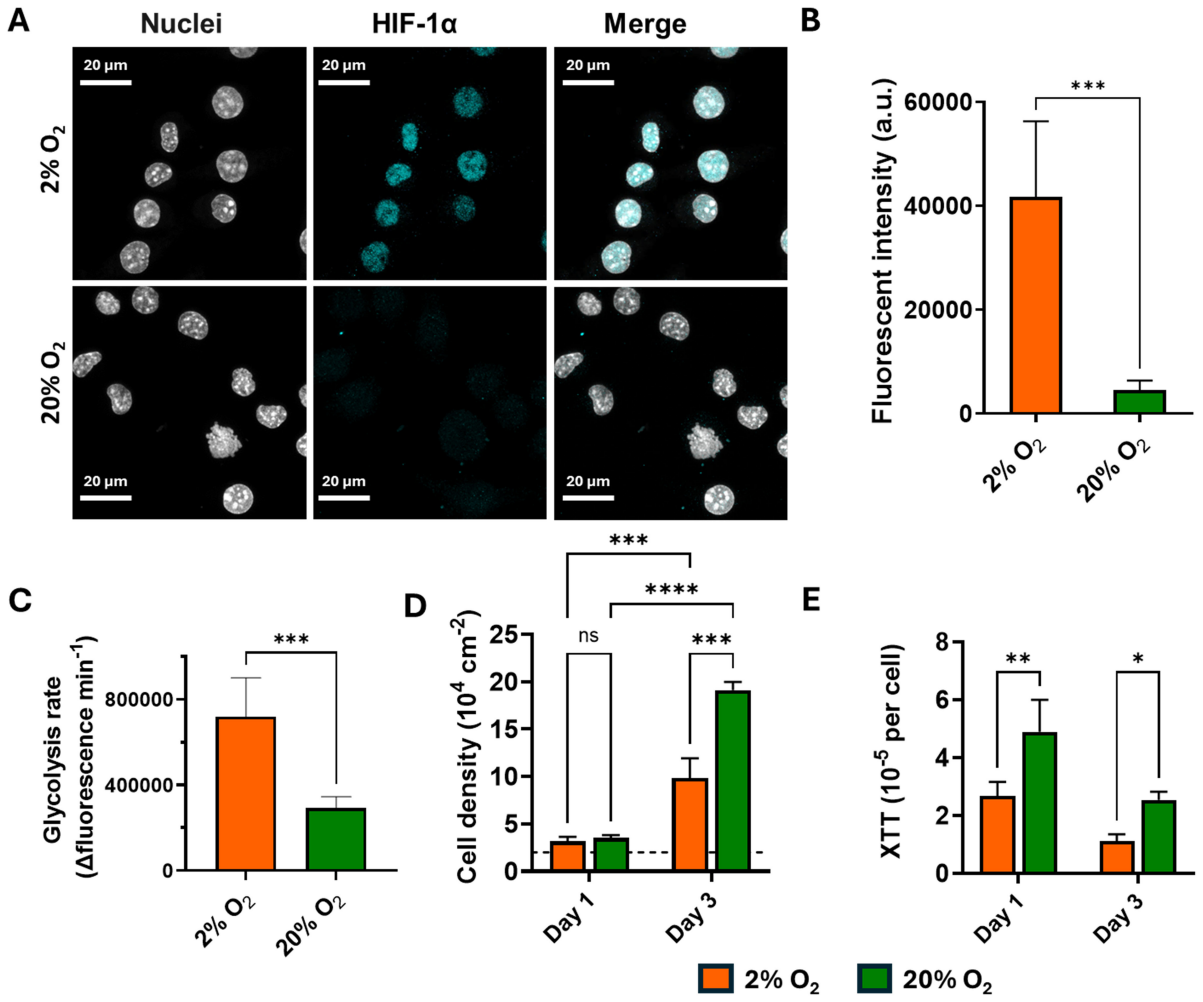
Macrophage (J774A.1) hypoxia-inducible factor-1 alpha (HIF-1α) activation, metabolic activity, and proliferation cultured under hypoxic (2% O_2_) and normoxic (20% O_2_) conditions. **(A)** Confocal laser scanning microscopy images of immunofluorescence stained hypoxia-inducible factor (HIF-1α) in the macrophage nucleus with anti-HIF-1α (*cyan*) and nuclei stained with DAPI (*gray*). **(B)** Quantification of the fluorescence intensities of HIF-1α in the nuclei shown in **(A)**, analyzed using Fiji software. **(C)** Glycolysis of the macrophages cultured under 2% or 20% O_2_ at 37°C measured by extracellular acidification in the glycolysis process. **(D)** Cell densities of the macrophages calculated using the Bürker–Türk counting chamber with trypan blue staining. The *black dotted line* indicates the initial seeding density. **(E)** Relative XTT per cell cultured under 2% and 20% O_2_ conditions for 1 and 3 days. The cell numbers were calculated from the microscope images. Student’s *t*-test was used for comparison of the fluorescence intensities, and two-way ANOVA followed by Tukey’s test was used for multiple group comparisons. *p < 0.05, **p < 0.01, ***p < 0.001, ****p < 0.0001, ns = not significant. Independently repeated experiments (*n* = 3).

HIF-1α activation in macrophages was observed in the nuclei of macrophages cultured for 24 h under the hypoxic condition (2% O_2_ tension), but not in the macrophages cultured under the normoxic condition (20% O_2_ tension), as shown in **Figures 1A, B**. The glycolysis rate of the macrophages showed a significantly increased glycolytic activity under hypoxia (2% O_2_), as indicated by the elevated extracellular acidification (**Figure 1C**). Macrophages proliferated under both hypoxic and normoxic conditions. However, the cell density after 3 days was significantly lower under hypoxia compared with that in normoxia (**Figure 1D**). The metabolic activity, assessed using the XTT assay, showed that the metabolic activity per cell was approximately twofold lower for hypoxia compared with normoxia (**Figure 1E**), which aligns with the glycolysis data (**Figure 1C**). Under the hypoxic condition, macrophages exhibited a more rounded morphology compared with the elongated morphology observed under normoxic conditions (**Supplementary Figure S2**).

### 3.2 Macrophage migration and morphology grown under hypoxic condition

**Figure 2:**
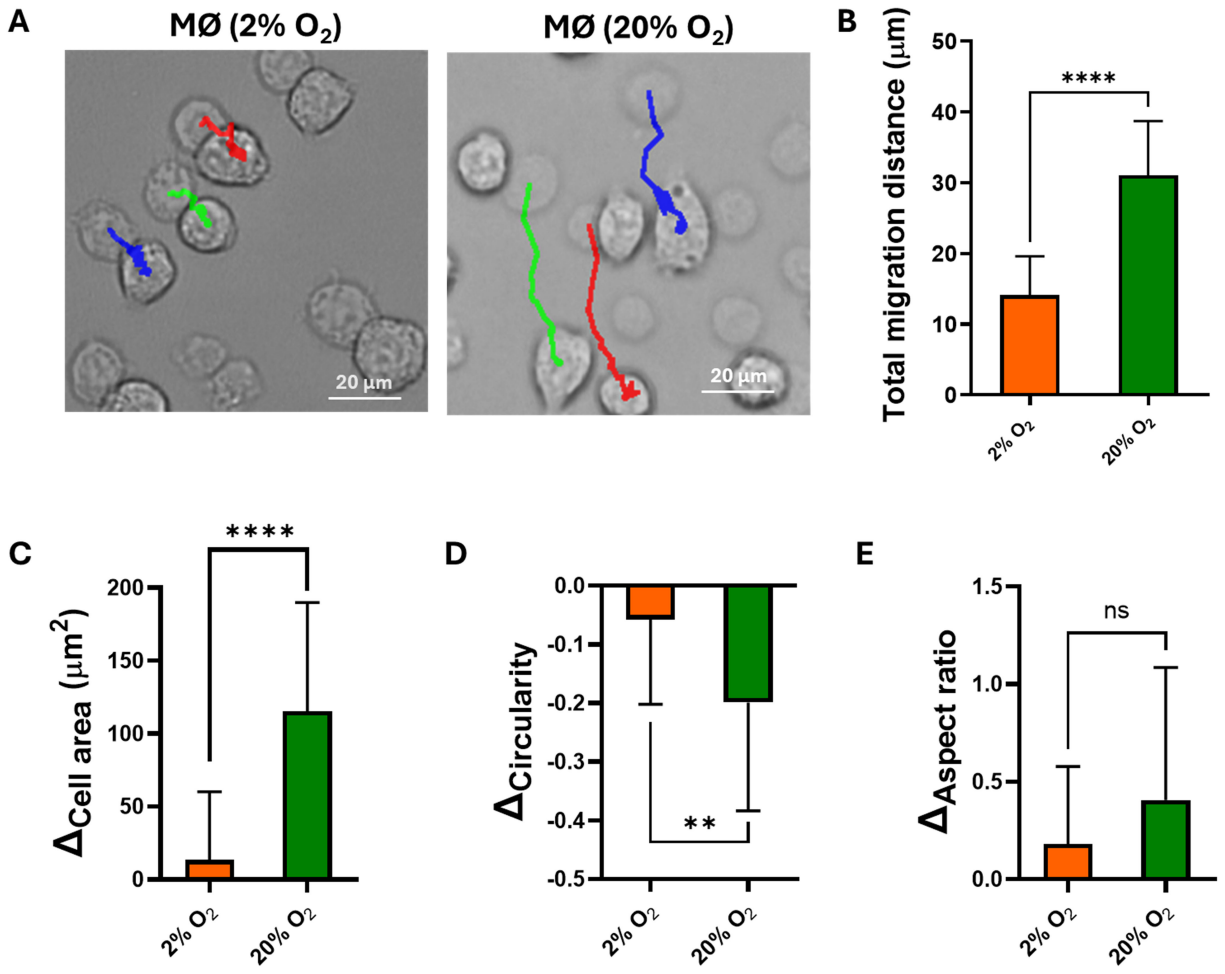
Live cell tracking of the macrophages (MØ) in 2% or 20% O_2_ with 5% CO_2_ at 37°C for 2 h. **(A)** Overlay of the time-lapse micrographs of the macrophages at the start of tracking (*half-transparent*, *t*_0_) and at the end of tracking (*t*_2h_). The *red*, *green*, and *blue lines* represent the macrophage migration tracking during 2 h. **(B)** Total migration distance of the macrophages over 2-h tracking in 2% or 20% O_2_. **(C)** The same as **(B)**, but for the changes in cell area. **(D)** The same as **(B)**, but for the changes in circularity. **(E)** The same as **(B)**, but for the changes in the aspect ratio of the macrophages. Student’s *t*-test was used for comparisons of the total migration distance. **p < 0.01, ****p < 0.0001. *ns*, not significant. Independently repeated experiments (*n* = 3).

The migration of macrophages under the hypoxic or normoxic condition was monitored in real time for 2 h using a microscope equipped with environmental controls. Representative images of the morphology of the macrophages under hypoxia and normoxia at the beginning (*t*_0_) and at the end (*t*_2h_) of live tracking are shown in **Figure 2A**, with their migration paths indicated by colored lines. Under the hypoxic condition (2% O_2_), the macrophages exhibited significantly shorter migration distances (14.1 ± 5.5 μm) over the 2-h period compared with those under normoxia, which migrated over a distance of 31.0 ± 7.7 μm (**Figure 2B**). Moreover, the extent of the morphological changes between the start (*t*_0_) and the end (*t*_2h_) of the tracking period was less pronounced under hypoxia than under normoxia in terms of cell area and circularity (**Figures 2C, D**), while the aspect ratio did not show any significant difference. Live tracking videos of the macrophages in 2% and 20% O_2_ are shown in **Supplementary Videos S1** and **S2**. It can be noted that the macrophages kept their round shape or round shape with blebbing under the hypoxic condition, in contrast to the elongated morphology observed under normoxia (**Supplementary Figure S3**).

### 3.3 Macrophages’ response to bacterial challenges

**Figure 3:**
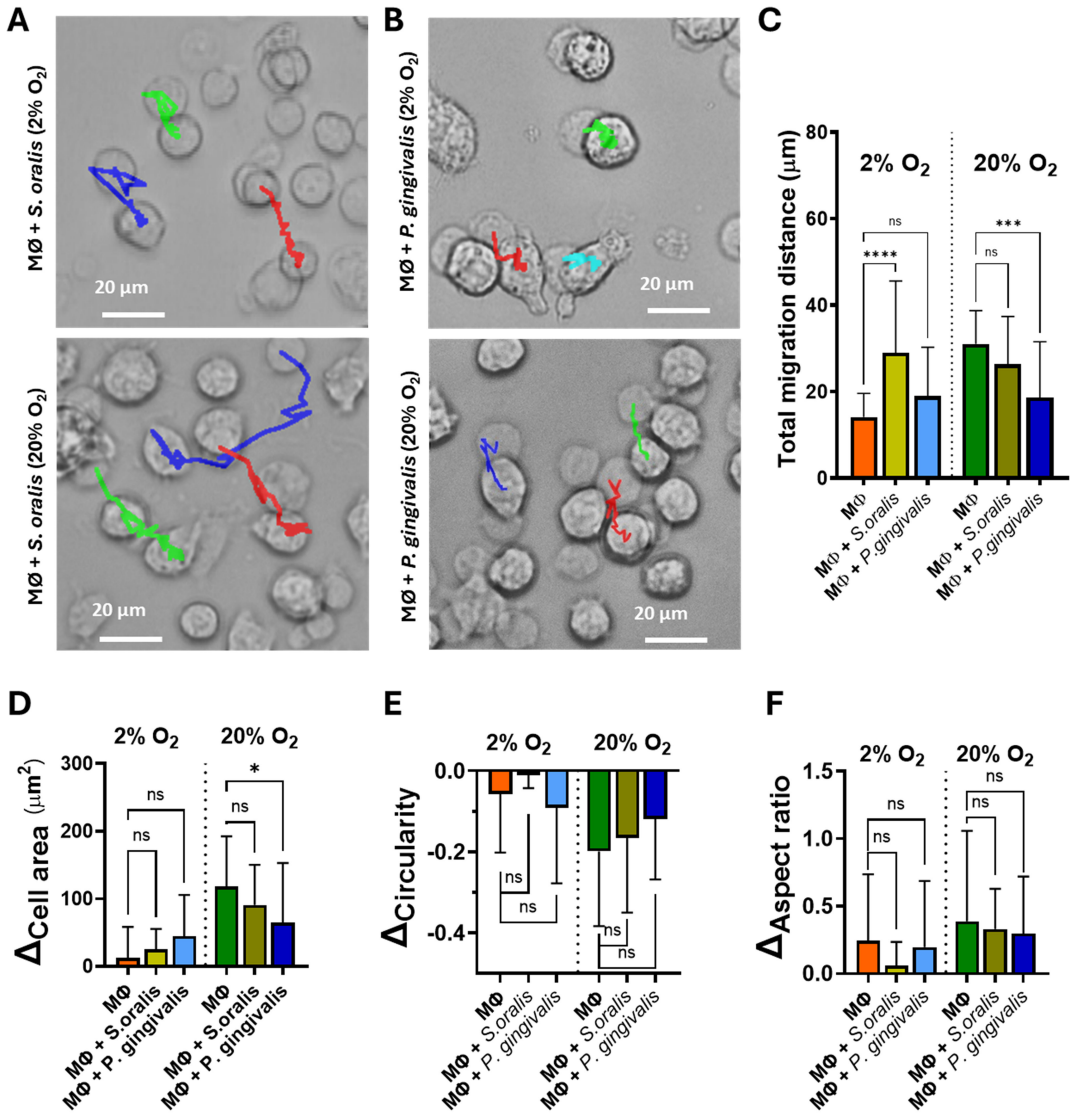
Live cell tracking of the macrophages (MØ) in the absence or presence of bacteria in 2% or 20% O_2_ with 5% CO_2_ at 37°C for 2 h. **(A)** Overlay of the time-lapse micrographs of the macrophages co-cultured with *Streptococcus oralis* at the start of tracking (*half-transparent*, *t*_0_) and at the end of tracking (*t*_2h_). *Red*, *green*, and *blue lines* represent the macrophage migration tracking during 2 h. **(B)** The same as **(A)**, but of macrophages in the presence of *Porphyromonas gingivalis.***(C)** Total migration distance of the macrophages over the 2-h tracking in 2% or 20% O_2_. **(D)** Changes in the cell area of macrophages after bacterial challenge from the start and the end of tracking, **(E)** The same as **(D)**, but for circularity. **(F)** The same as **(D)**, but for the aspect ratio. One-way ANOVA followed by Tukey’s test was used for multiple group comparisons. *p < 0.05, ***p < 0.001, ****p < 0.0001. *ns*, not significant. Independently repeated experiments (*n* = 3).

To investigate the response of macrophages to microenvironmental stimuli, they were challenged with the oral bacteria *S. oralis* ATCC 35037 and *P. gingivalis* ATCC 33277 under optimized co-culture conditions. The presence of *S. oralis* under hypoxia significantly enhanced macrophage migration, but the migration under the normoxic condition did not change (**Figures 3A, C**). In contrast, the total migration distance of the macrophages under hypoxia for *P. gingivalis* was not affected, but the migration distance was reduced under the normoxic condition (**Figures 3B, C**). The morphology of the macrophages, expressed as cell area, circularity, and aspect ratio, remained unchanged under hypoxic and normoxic conditions regardless of the bacterial strain (**Figures 3D–F**, **Supplementary Videos S3**-**S6**); however, *P. gingivalis* decreased the macrophage cell area under the normoxic condition.

### 3.4 Macrophages limit intracellular bacterial growth under hypoxia

**Figure 4:**
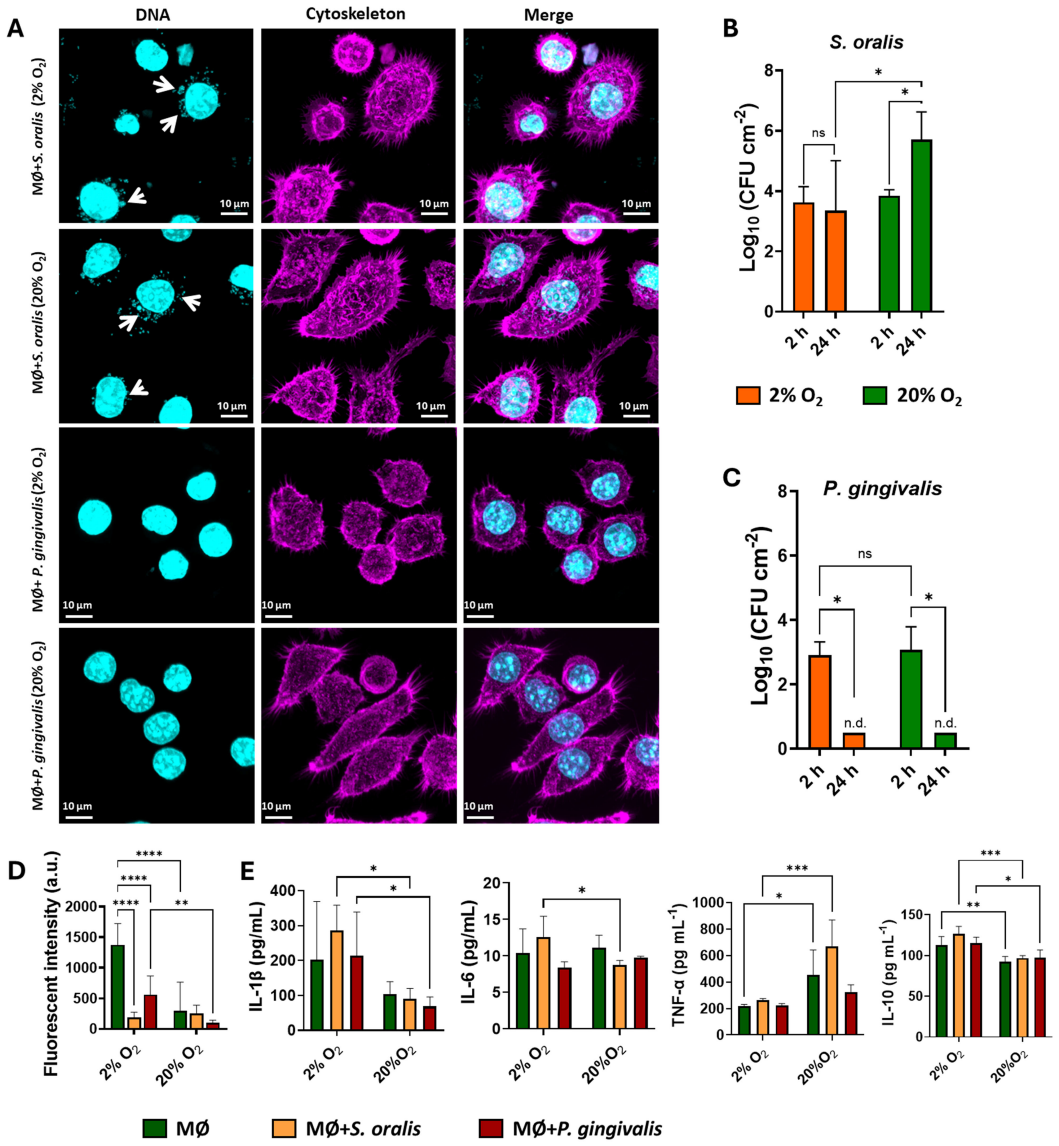
Macrophages (MØ) co-cultured with *Streptococcus oralis* or *Porphyromonas gingivalis* in 2% or 20% O_2_ with 5% CO_2_ at 37°C for 2 or 24 h. **(A)** Confocal laser scanning microscopy (CLSM) images of the macrophages co-cultured with *S. oralis* or *P. gingivalis* under different O_2_ conditions for 2 h, stained with DAPI (DNA, *cyan*) and phalloidin (cytoskeleton, *magenta*). *White arrows* point to the intracellular bacteria. **(B)** Intracellular *S. oralis* after co-culture with macrophages in 2% and 20% O_2_ for 2 or 24 h, detected as colony-forming units (CFU) enumerated via plating on agar plates. **(C)** The same as **(B)**, but for *P. gingivalis*. *n.d.*, non-detectable (detection limit, 10^2^ CFU cm^−2^). **(D)** Intracellular reactive oxygen species (ROS). **(E)** Cytokines produced by the macrophages with or without *S. oralis* and *P. gingivalis* in 2% or 20% O_2_ with 5% CO_2_ at 37°C co-cultured for 2 h. Intracellular ROS and the cytokine concentrations were quantified using Fiji software and ELISA kits, respectively. One-way or two-way ANOVA followed by Tukey’s test was used for multiple group comparisons. *p < 0.05, ** p<0.01, ***p < 0.001, ****<0.0001. *ns*, not significant. Independently repeated experiments (*n* = 3).

The CLSM images of the macrophages in the presence of bacteria for 2 h are shown in **Figure 4A**. Despite these morphological differences under different O_2_ tensions, the macrophages were capable of engulfing *S. oralis* and *P. gingivalis* bacteria during co-culturing for 2 h under both conditions (**Figure 4A**; intracellular bacteria indicated by white arrows). This was consistent with the CFU, which revealed no significant difference in the number of intracellular bacteria under 2% and 20% O_2_ (**Figures 4B, C**). Note that the extracellular bacteria were eradicated using gentamicin treatment after 2 h.

When macrophages were co-cultured with *S. oralis* for 24 h, there was a significant increase in intracellular bacteria under the normoxic condition (20% O_2_). Moreover, an initial biofilm of *S. oralis* was observed (**Supplementary Figure S4**) on apoptosis of the macrophages after 24 h of co-culture. However, there were much less intracellular *P. gingivalis* left after co-culturing with the macrophages for 24 h, with the number dropping below the CFU detection limit (**Figure 4C**), which indicated effective killing of *P. gingivalis* by the macrophages regardless of the normoxic or hypoxic condition. The macrophages co-cultured with *P. gingivalis* became elongated after 24 h (**Supplementary Figure S4**), in contrast to the macrophages cultured without bacteria for 24 h under the hypoxic condition (**Supplementary Figure S3**).

The intracellular ROS production dramatically increased in macrophages under 2% O_2_ level compared with 20% O_2_ level (**Figure 4D**, **Supplementary Figure S5**). However, when the macrophages were exposed to *S. oralis* and *P. gingivalis*, the intracellular ROS decreased under both hypoxic and normoxic conditions compared with the macrophages without bacteria. The cytokine secretion by macrophages was modulated under hypoxic conditions. The production of IL-1β and IL-10 increased following hypoxia exposure, whereas the levels of TNF-α decreased compared with that in the normoxic condition. Among the examined cytokines, only the expression of TNF-α was significantly influenced by the bacterial challenges (**Figure 4E**). In contrast, the secretion of IL-6 remained very similar regardless of the oxygen levels.

## 4 Discussion

**Figure 5:**
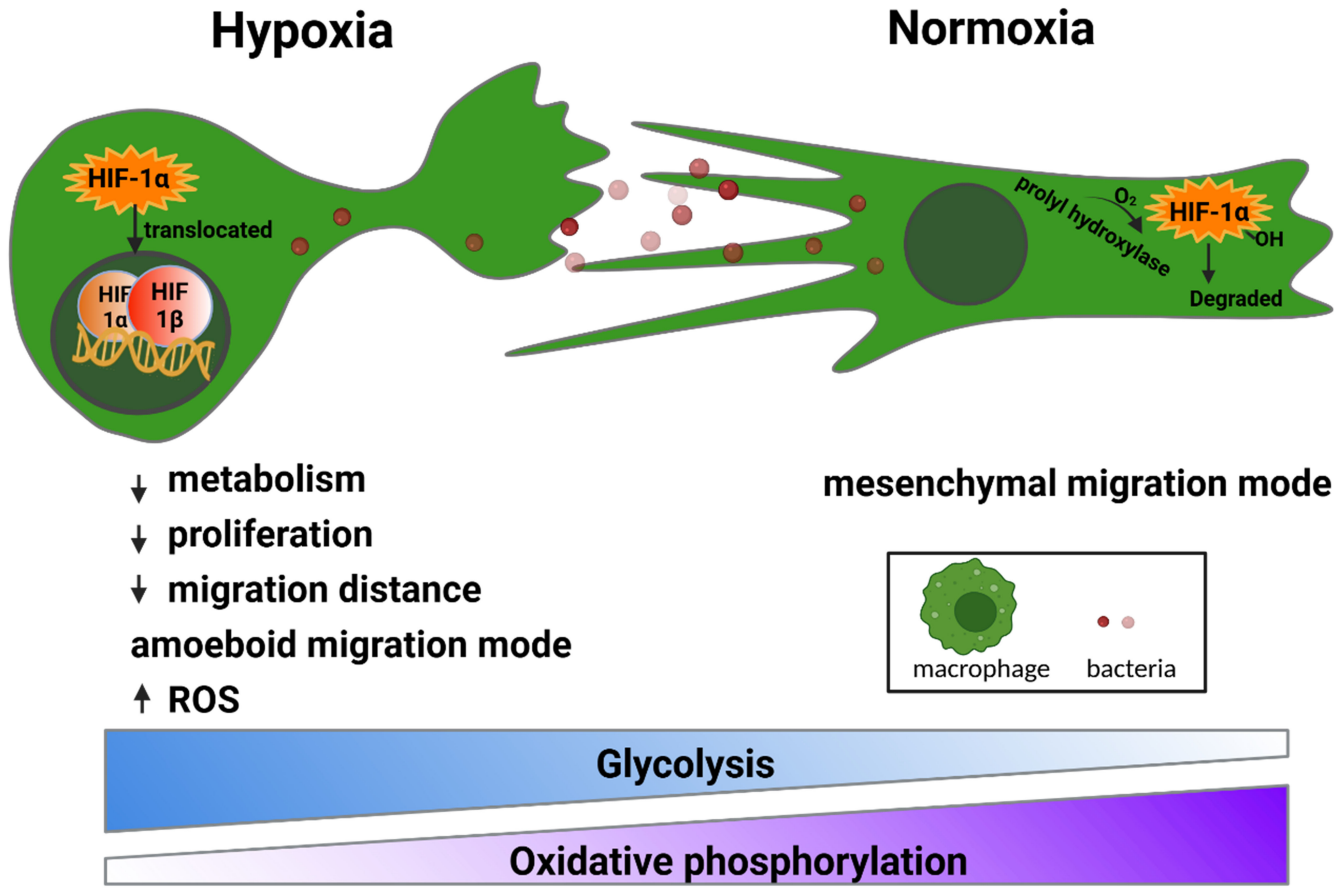
Schematic comparison of the macrophage adaptations under hypoxia and normoxia. Under the hypoxic condition, the macrophages exhibited reduced metabolism and proliferation, shorter migration distances with a transition to an amoeboid migration mode, and increased dependence on glycolysis compared with the macrophages under the normoxic condition.

This study demonstrated that physiologically relevant hypoxia (2% O_2_) significantly modulates the behavior of macrophages, influencing key cellular processes such as proliferation, migration, cytokine production, and antimicrobial response. These effects are mediated primarily through the stabilization and nuclear translocation of HIF-1α, which governs the adaptive shift from oxidative phosphorylation to glycolysis (**Figure 5**).

As the central regulator of oxygen sensing, the HIF-1α pathway is the key player in cell adaptation to different oxygen tensions (Taylor and Scholz, 2022; Kierans et al., 2023). Upon successful translocation into the nuclei, HIF-1α triggers a series of changes, including metabolism, surface adhesion, proliferation, and pro-inflammatory responses (Taylor and Scholz, 2022). Under the hypoxic condition (2% O_2_), HIF-1α was activated in the macrophage nuclei, while its expression was minimal in both the cytoplasm and the nuclei under the normoxic condition (**Figures 1A, B**). This is due to the short half-life of HIF-1α, i.e., 3–6 min in the cytoplasm, which is rapidly hydroxylated by prolyl hydroxylase in the presence of oxygen and quickly degraded by proteasome in the cytoplasm (Moroz et al., 2009). One of the immediate functional consequences of this metabolic shift is a reduction in macrophage proliferation. Under the hypoxic condition, we observed a 45% decrease in cell density (**Figure 1D**), likely due to a glycolytic switch under this condition (**Figure 1C**) and the consequent decline in aspartate production—an essential component for nucleotide synthesis and cell proliferation (Birsoy et al., 2015). Pharmacological inhibitors such as YC-1 and PX-478, or the siRNA-mediated silencing of HIF-1α, have been shown to downregulate glycolytic reprogramming and immunoregulation (Kong et al., 2023; Liang et al., 2025).Live cell imaging revealed a reduction in the migration distance under the hypoxic condition (2% O_2_) (**Figure 2**), which is due to the activation of the migration inhibition factor under hypoxia (Fu et al., 2010; Safi et al., 2020). Under the normoxic condition, the macrophages primarily used a mesenchymal-like mode to migrate, characterized by elongated shapes with the formation of filopodia (**Supplementary Video S1**). In contrast, under the hypoxic condition (2% O_2_), the macrophages transitioned into an amoeboid-like mode of migration, characterized by more rounded or ellipsoid shapes (**Supplementary Video S2**). This phenotypic shift is driven by hypoxia-induced downregulation of the adhesion molecules and cytoskeleton rearrangement (Te Boekhorst et al., 2022). Amoeboid migration, largely being adhesion-independent, gave the macrophages a Brownian-type movement, which is a more energy-efficient mode that allows macrophages to respond more rapidly but less directionally. This Brownian movement leads to shorter migration distances under hypoxic conditions (Liu et al., 2020; Parlani et al., 2023).

In addition to morphological and metabolic adaptation, hypoxia also drives an increase in intracellular ROS (**Figure 4D**), likely due to the impaired mitochondrial metabolism resulting in increased ROS production as an adaptive redox signal (Okoye et al., 2023). Cytokine expression revealed mixed inflammatory and regulatory phenotypes under hypoxia, with increased IL-1β and IL-10 and reduced TNF-α under 2% O_2_ (**Figure 4E**). HIF-1α activation enhanced the IL-1β expression, while the TNF-α expression in macrophages decreased under hypoxia (Ogryzko et al., 2019; Chen et al., 2023). Together, these changes reflect a nuanced reprogramming of macrophage function in response to low oxygen levels, whereby macrophages simultaneously drive antimicrobial defense (via IL-1β) and attempt to limit excessive inflammation (via IL-10).

To further explore the responses of macrophages under hypoxia, we investigated their interaction with oral bacteria. Under the hypoxic condition, the macrophages migrated longer distances when exposed to *S. oralis* compared with conditions without *S. oralis* or with *P. gingivalis* (**Figure 3**). Despite this difference in migration, the phagocytosis of *S. oralis* was comparable between hypoxia and normoxia after 2 h (**Figures 4A, B**). Notably, the observed decrease in the intracellular ROS levels in the macrophages with *S. oralis* can be attributed to the ability of the bacterium to produce hydrogen peroxide (H_2_O_2_). This acts as a signal that triggers the activation of the antioxidant defense system in macrophages, particularly the Nrf2 pathway (Tang et al., 2022). The activation of Nrf2 leads to the upregulation of antioxidant enzymes, which not only neutralize the H_2_O_2_ produced by *S. oralis* but also scavenge the intracellular ROS, resulting in a net decrease in the intracellular ROS levels (**Figure 4D**).

*P. gingivalis* triggered a different macrophage response by activating Toll-like receptor 2 (TLR2) signaling and cytoskeletal rearrangement. *P. gingivalis* suppressed the migration of macrophages (Angabo et al., 2024; Polara et al., 2024), which was evident under normoxia after 2 h (**Figure 3C**). In addition, intracellular *P. gingivalis* was eliminated after 24 h of co-culture under both hypoxic and normoxic conditions (**Figure 4C**). Similarly to *S. oralis*, *P. gingivalis* also suppresses the ROS production (**Figure 4D**) to survive and, in the end, promotes a chronic inflammatory state (Chopra et al., 2020).

These findings underscore the complexity of phagocytosis in host–pathogen interactions. While phagocytosis is a critical step in the elimination of infection, it does not mark the complete resolution of infection (Okahashi et al., 2016). In some cases, phagocytosis provides a replicative niche for these intracellular bacteria, such as *S. oralis* in this study (**Supplementary Figure S4**). This nutrient-rich niche protects intracellular bacteria from surveillance and antimicrobials, and allowing these bacteia to form biofilms (Price and Vance, 2014; Mitchell et al., 2016). Upon macrophage death, these intracellular bacteria can be released, spreading to other surfaces and cells (Mahamed et al., 2017; Vidakovic et al., 2023).

Our findings emphasize the remarkable plasticity of macrophages—their ability to reprogram metabolic and functional profiles in response to environmental cues such as oxygen tension. Under hypoxia, the macrophages exhibited a hybrid phenotype with enhanced glycolysis, altered cytokine expression, and increased ROS production and maintained phagocytic capacity. Such functional flexibility is vital in complex tissue microenvironments such as inflamed periodontal pockets, where simultaneous pro-inflammatory and regulatory activities are required. A better understanding of this plasticity could inform therapeutic strategies that fine-tune the macrophage responses to enhance host defense without exacerbating tissue damage. Such strategies, including oxygen-releasing therapy or hypoxia-responsive drug delivery, have been developed to improve infection control and restore the surrounding tissues (Su et al., 2022; Müller-Heupt et al., 2023; Ming et al., 2024). Similar oxygen-modulating interventions have shown promising outcomes in other diseases, such as infected wounds (Huang et al., 2024) and diabetic ulcers (Zhuge et al., 2025). In these conditions, localized oxygen delivery restores tissue normoxia, enhances macrophage polarization toward a reparative phenotype, promotes angiogenesis, and mitigates chronic inflammatory responses, collectively supporting tissue regeneration. Furthermore, modulating macrophage polarization to a pro-inflammatory phenotype can boost the antimicrobial outcomes in the eradication of bacterial biofilms (Liu et al., 2019; Wu et al., 2023). In this study, the single macrophage cell line and the constant 2% oxygen environment represent a limitation as they cannot fully replicate the dynamic, multicellular nature of inflamed tissues. Nonetheless, they provide a controlled and physiologically relevant framework for delineating the adaptations of macrophages to hypoxia.

## 5 Conclusion

Taken together, our findings highlight the remarkable plasticity of macrophages and their ability to adapt to their metabolism, migration mode, and phagocytosis in response to oxygen tensions via HIF-1α activation. These adaptive mechanisms are crucial for the development of targeted therapies aimed at modulating macrophage activity and inhibition or the killing of intracellular bacteria.

## Data Availability

The original contributions presented in the study are included in the article/**Supplementary Material**. Further inquiries can be directed to the corresponding authors.
