# Supplementary material for: Macrophage adaptation to hypoxia: metabolism, migration, and phagocytosis
Source: Front Cell Infect Microbiol. 2025 Dec 12;15:1706664. doi: 10.3389/fcimb.2025.1706664 (PMC12740870; doi:10.3389/fcimb.2025.1706664)
Supplement: Supplementary file 1 [file DataSheet1.pdf]

Supplementary

Macrophage Adaptation to Hypoxia: Metabolism, Migration, and Phagocytosis

Lu Yuan*, Tiemen Mellema, Gésinda I. Geertsema-Doornbusch, Henny C. van der Mei*

University of Groningen and University Medical Center Groningen, Biomaterials and Biomedical Technology, 9713 AV Groningen, The Netherlands

Corresponding author at: University of Groningen and University Medical Center Groningen, Biomaterials and Biomedical Technology, 9713 AV Groningen, The Netherlands

Email address: l.yuan@umcg.nl; h.c.van.der.mei@umcg.nl

Supplementary figure legends

Supplementary Figure S1. Optimization of the co-culture medium for macrophage J774A.1, and *S. oralis* ATCC 35037 and *P. gingivalis* ATCC 33258. The co-culture medium was developed using complete cell culture media (DMEM) with two glucose concentrations: 4.5 g L^{-1} (DMEM-HG) and 1 g L^{-1} (DMEM-LG), each supplemented with 10% fetal bovine serum. These media were mixed with bacterial broth (BHI supplemented with 1 g L^{-1} yeast extract) at varying volume ratios, from 100% to 0% DMEM. (**A**) Macrophages (initial seeding 5×10^3 macrophages per well in a 96-well plate) were cultured in 100% DMEM-LG, 90% DMEM-LG mixed with 10% BHI, and 60% DMEM-LG mixed with 40% BHI, and incubated (5% CO_2, 95% air, 37°C) for 1 and 3 days. (**B**) The metabolic activity of macrophages in different media was assessed using the XTT assay. (**C)** *S. oralis* ($1*10^6$ mL^{-1}) inoculated in the different growth media and cultured in an incubator (5% CO_2, 95% air) at 37°C for 6 h; followed by CFU to estimate viable bacteria (**D**) *P. gingivalis* were inoculated at a density of $1*10^8$ mL^{-1} to the different growth media and anaerobically cultured for 48 h, with CFU used to estimate viable bacteria. (**E**) *S. oralis* were inoculated at a density of $1*10^6$ mL^{-1} to the optimized medium (80%DMEM-LG+10% FBS+ 10%BHI) and cultured under different O_2 levels (0%, 2% and 20% O_2) for 6 h, with CFU used to estimate viable bacteria. (**F**) *P. gingivalis* were inoculated at a density of $1*10^8$ mL^{-1} to the optimized medium (80%DMEM-LG+10% FBS+ 10%BHI) and cultured under different O_2 levels (0%, 2% and 20% O_2) for 48 h, with CFU used to estimate viable bacteria. Dotted lines represent the initial bacterial inoculation density.

There was no significant difference between DMEM-LG and DMEM-HG regarding the morphology, metabolic activity, or proliferation of the macrophages. The optimal DMEM concentration for culturing macrophages was between 80-100%. However, the rapidly growing *S. oralis* caused a pH change in DMEM-HG (95%–60% DMEM-HG), as indicated by the yellow color shift of phenol red, whereas DMEM-LG remained pH stable. The optimal DMEM concentration range was 0%–95% for *S. oralis* and 0%–100% for *P. gingivalis*. Based on these results, 90% DMEM-LG mixed with 10% BHI was selected as the optimal co-culture medium.

Supplementary Figure S2. Phase contrast microscopic images of macrophages J774A.1 cultured under 2% O_2 and 20% O_2 for 1 and 3 days.

Supplementary Figure S3. Macrophages (MØ) cultured in 2% O_2 or 20% O_2 with 5% CO_2 at 37°C for 2 h and 24 h. CLSM images of macrophages stained with DAPI (DNA, cyan) and phalloidin (cytoskeleton, magenta).

Supplementary Figure S4. CLSM images of macrophages (MØ) co-cultured with *S. oralis* (10^7 mL^{-1}) or *P. gingivalis* (10^7 mL^{-1}) in 2% O_2 or 20% O_2 with 5% CO_2 at 37°C for 24 h; DNA and cytoskeleton stained by DAPI (cyan) and phalloidin (magenta), respectively. Note, macrophages co-cultured with *S. oralis* for 24 h exhibited severe

damage which results in little presence of cytoskeleton under both hypoxia and normoxia conditions.

Supplementary Figure S5. Intracellular ROS of macrophages (MØ) cultured with or without *S. oralis* (10^7 mL^{-1}) or *P. gingivalis* (10^7 mL^{-1}) in (A) 2% O_2 or (B) 20% O_2 with 5% CO_2 at 37°C for 2 h; DNA and intracellular ROS stained by Hoescht 33342 (blue) and DCFH-DA (green), respectively.

Supplementary Movie S1. Live-cell tracking of macrophages in 2% O_2 for 2 h

Supplementary Movie S2. Live-cell tracking of macrophages in 20% O_2 for 2 h

Supplementary Movie S3. Live-cell tracking of macrophages co-cultured with *S. oralis* in 2% O_2 for 2 h

Supplementary Movie S4. Live-cell tracking of macrophages co-cultured with *S. oralis* in 20% O_2 for 2 h

Supplementary Movie S5. Live-cell tracking of macrophages co-cultured with *P. gingivalis* in 2% O_2 for 2 h

Supplementary Movie S6. Live-cell tracking of macrophages co-cultured with *P. gingivalis* in 20% O_2 for 2 h
