# Supplementary figures and images for: Macrophage adaptation to hypoxia: metabolism, migration, and phagocytosis
Source: Front Cell Infect Microbiol. 2025 Dec 12;15:1706664. doi: 10.3389/fcimb.2025.1706664 (PMC12740870; doi:10.3389/fcimb.2025.1706664)

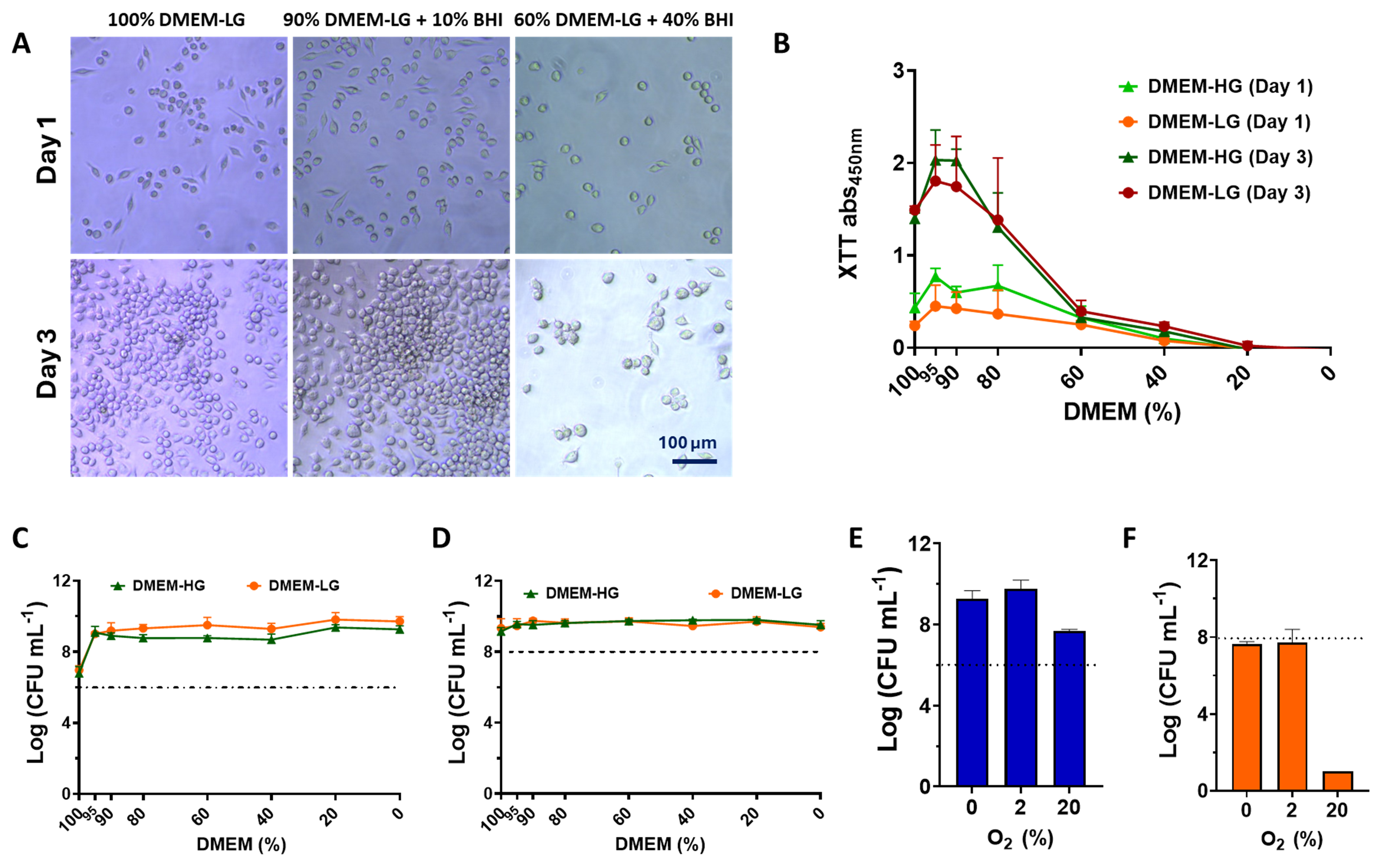

Supplement: Supplementary file 2 [file Image1.tif]

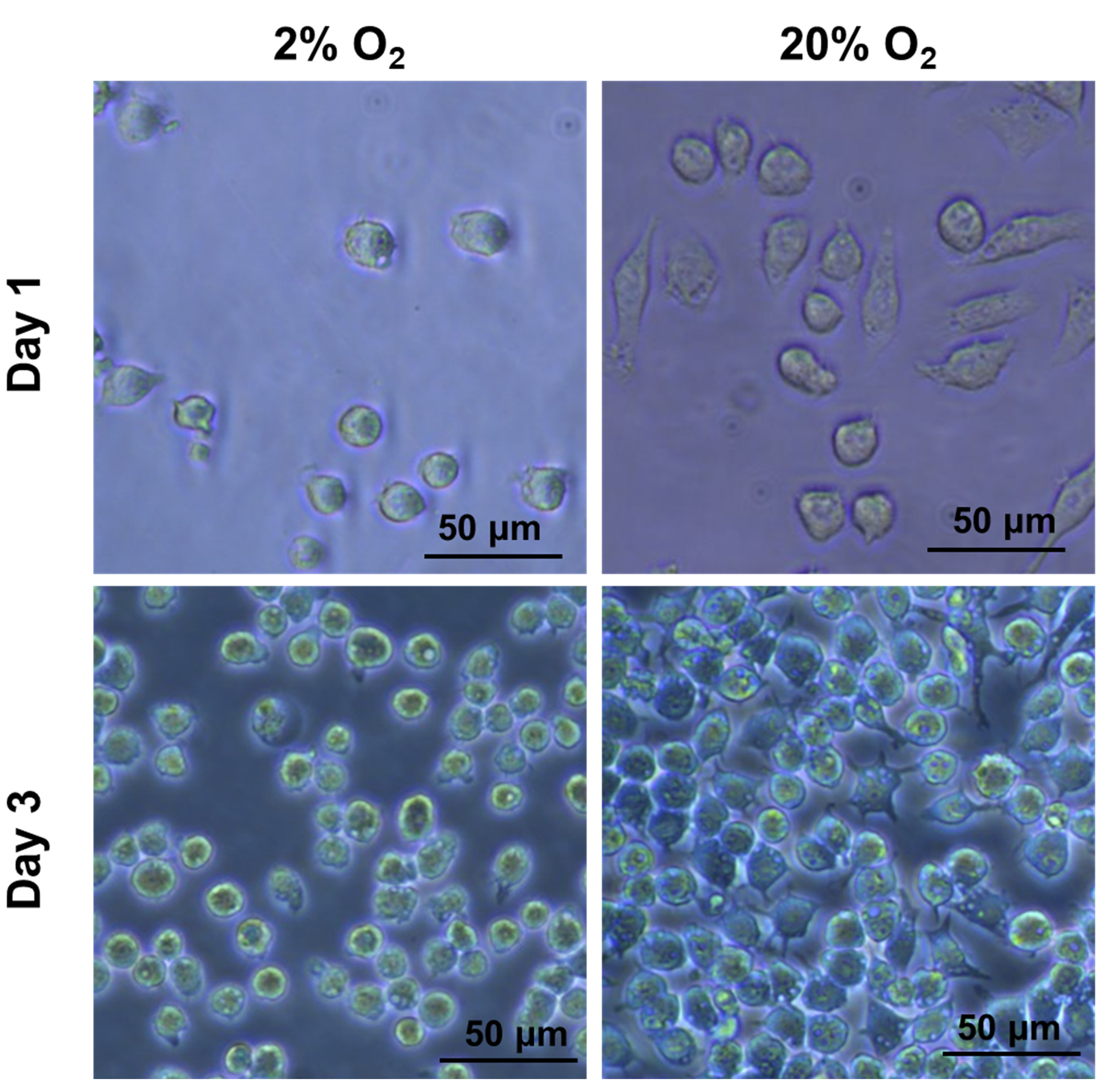

Supplement: Supplementary file 3 [file Image2.tif]

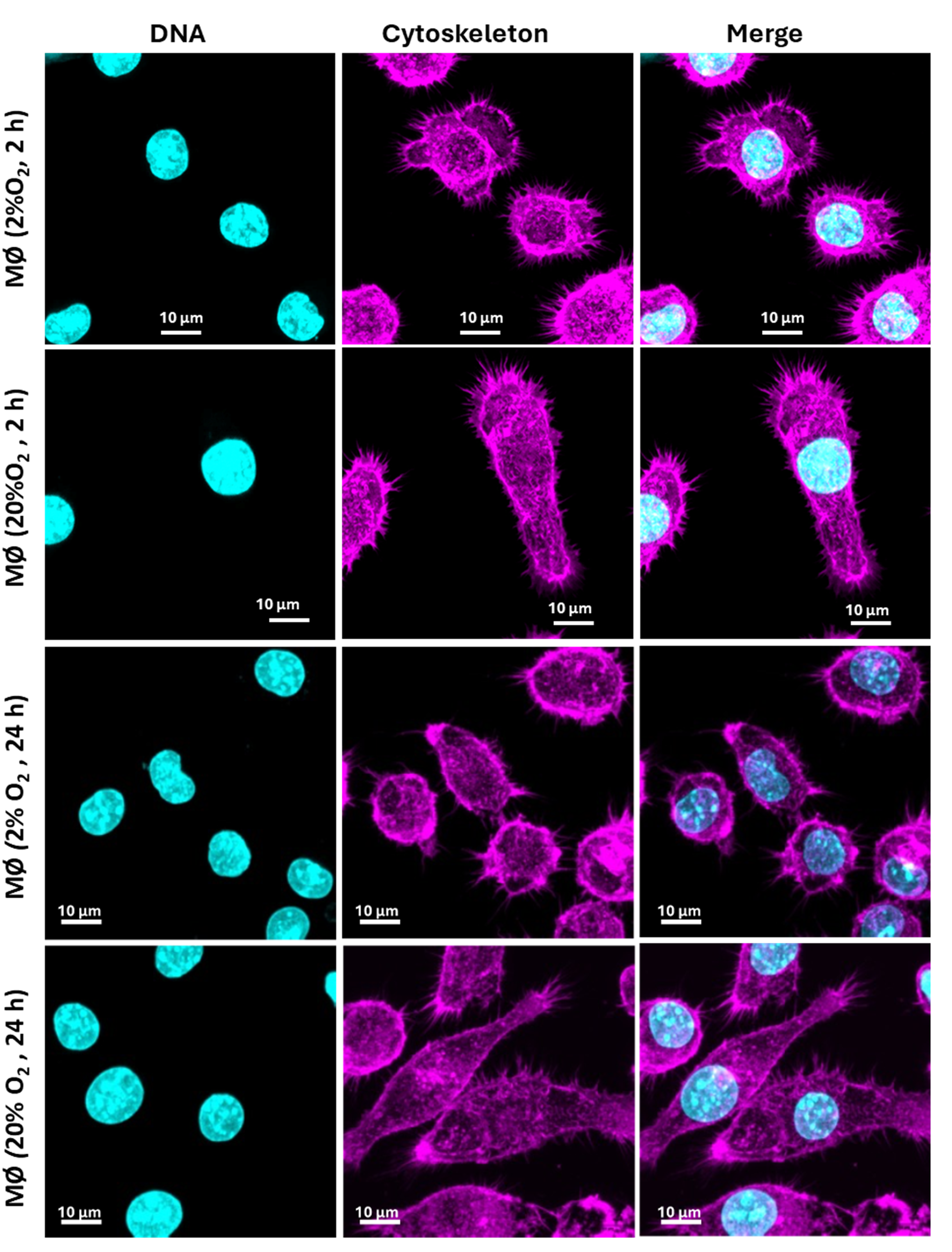

Supplement: Supplementary file 4 [file Image3.tif]

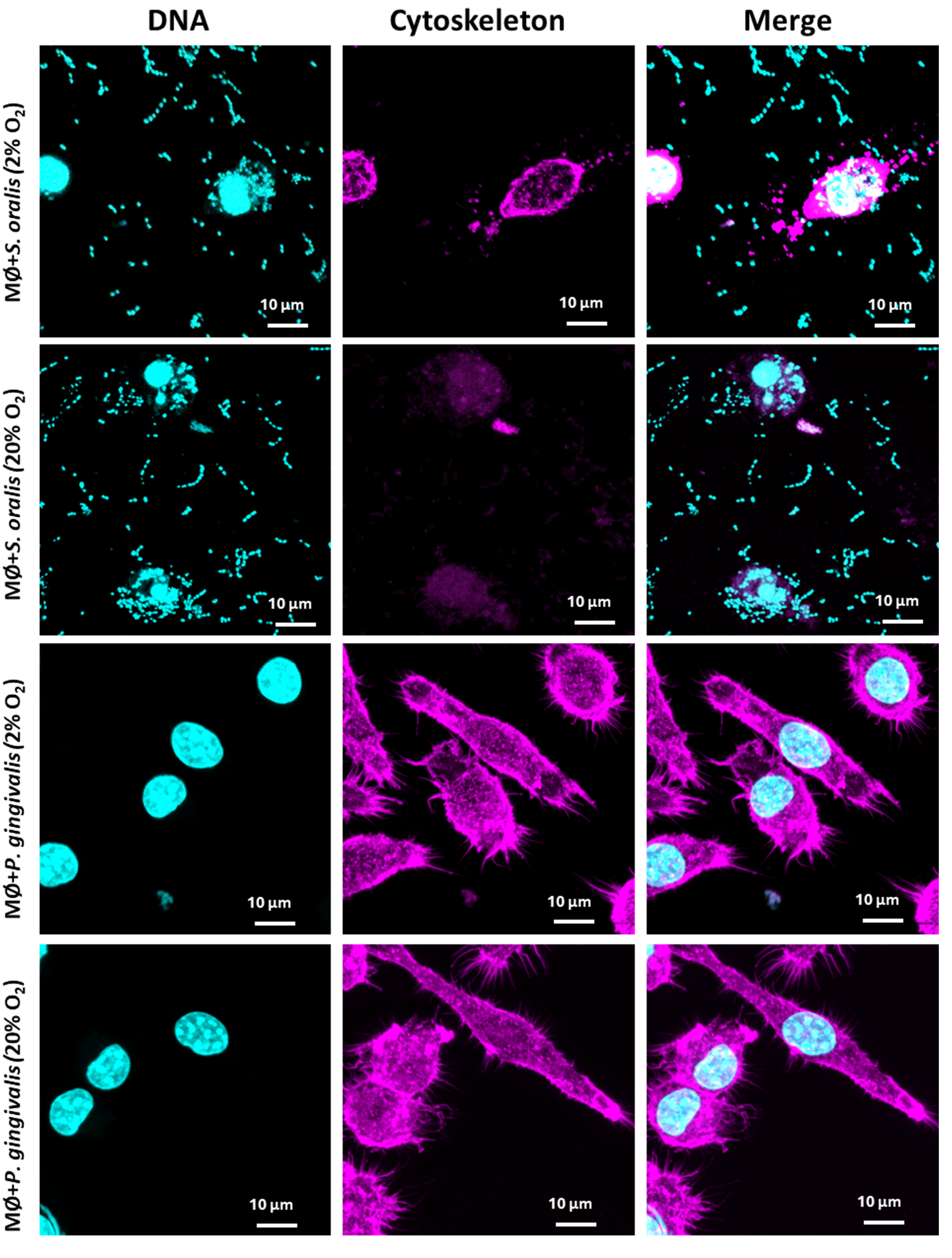

Supplement: Supplementary file 5 [file Image4.tif]

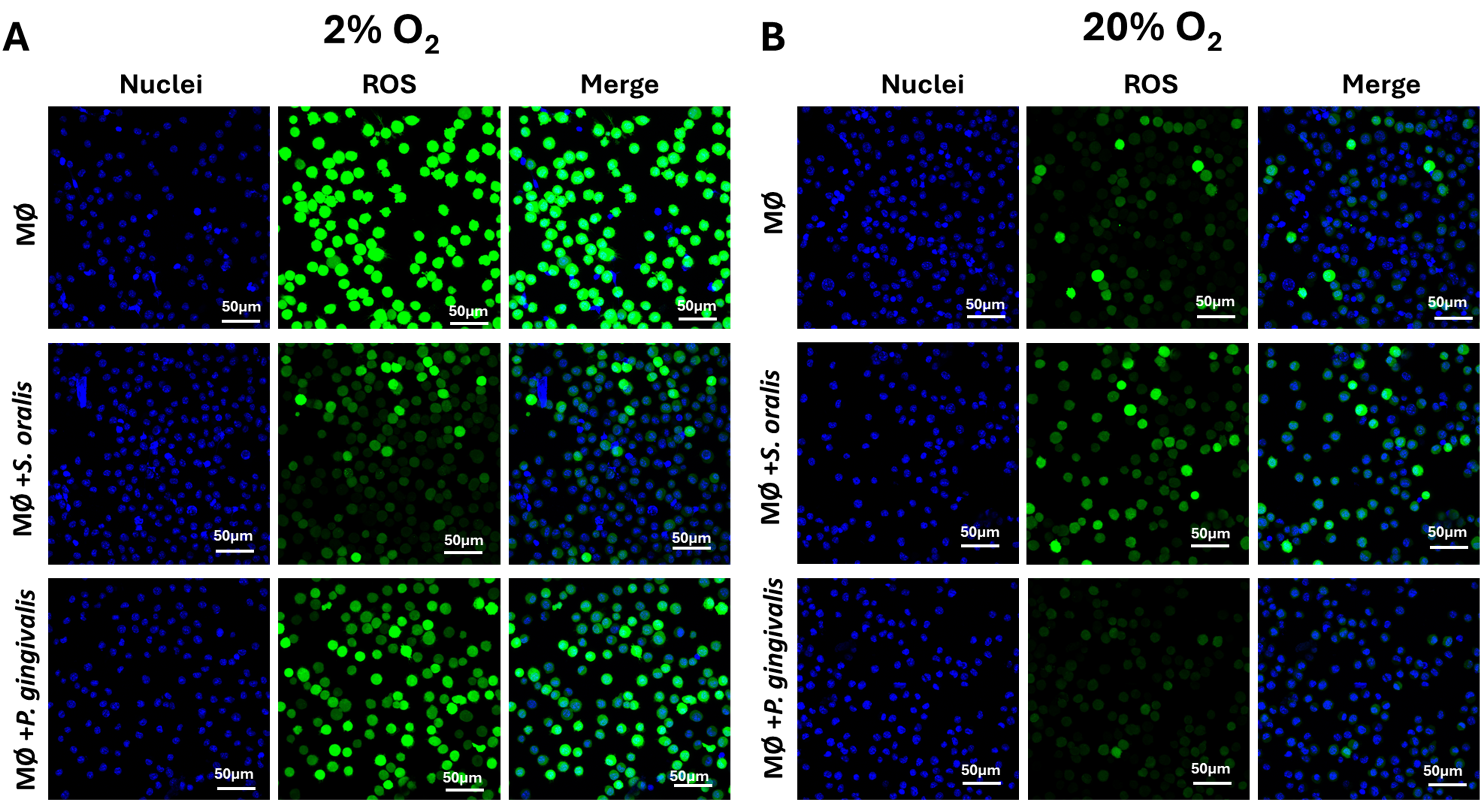

Supplement: Supplementary file 6 [file Image5.tif]
